# Prematurity and functional gastrointestinal disorders in infancy: a cross-sectional study

**DOI:** 10.1590/1516-3180.2021.0622.R1.29102021

**Published:** 2022-05-30

**Authors:** Marcela Montenegro Braga Barroso Gondim, Ana Lucia Goulart, Mauro Batista de Morais

**Affiliations:** I MD. Physician, Division of Pediatric Gastroenterology, Escola Paulista de Medicina (EPM), Universidade Federal de São Paulo (UNIFESP), São Paulo (SP), Brazil.; II MD, PhD. Physician and Associate Professor, Division of Neonatal Pediatrics, Escola Paulista de Medicina (EPM), Universidade Federal de São Paulo (UNIFESP), São Paulo (SP), Brazil.; III MD, PhD. Physician and Full Professor, Division of Pediatric Gastroenterology, Escola Paulista de Medicina (EPM), Universidade Federal de São Paulo (UNIFESP), São Paulo (SP), Brazil.

**Keywords:** Colic, Constipation, Gastroesophageal reflux, Diarrhea, infantile, Gastrointestinal diseases, Preterm newborn, Gastroenteropathies, Childhood diarrhea, Prematurity, Functional gastrointestinal disorders, Rome IV criteria

## Abstract

**BACKGROUND:**

Functional gastrointestinal disorders (FGIDs) are defined as a variable combination of chronic or recurrent gastrointestinal symptoms that are not explained by structural or biochemical abnormalities. Their relationship with prematurity has been increasingly studied.

**OBJECTIVE:**

To compare the frequency of FGIDs in preterm and term infants and to evaluate whether invasive procedures during the neonatal period in preterm infants are associated with greater likelihood of FGIDs in the first two years of life.

**DESIGN AND SETTING:**

Controlled nested cross-sectional study conducted in a Brazilian university hospital.

**METHODS:**

This was a controlled nested cross-sectional study on a retrospective cohort of infants born preterm who were compared with infants born at term regarding the presence of FGIDs. Medical consultations were conducted by a single pediatric gastroenterologist to obtain information on the gestational and neonatal periods and on clinical manifestations of the digestive tract. The Rome IV criteria for the diagnosis of FGIDs were used.

**RESULTS:**

A total of 197 infants (< 24 months), including 99 preterm and 98 term infants, were studied. Infant regurgitation was more prevalent in term infants (35.1% and 15.6%; P < 0.001). The frequencies of other FGIDs (infant colic, functional constipation, functional diarrhea and infant dyschezia) in preterm infants did not differ from those of term infants (P > 0.05). No relationship was found between invasive procedures during the neonatal period and development of FGIDs in preterm infants.

**CONCLUSION:**

Infants born preterm did not have higher frequency of FGIDs in the first two years of life.

## INTRODUCTION

Functional gastrointestinal disorders (FGIDs) are defined as a variable combination of chronic or recurrent gastrointestinal symptoms that are not explained by structural or biochemical abnormalities.^1^ In addition to the discomfort caused by clinical manifestations, FGIDs cause stress and anxiety in families. They compromise these families’ quality of life and cause them to incur increased healthcare expenditure.^2^ ^,^ ^3^

A review of the literature^4^ published in 2015 showed that the prevalence of FGIDs in infants shows wide variability due to the heterogeneity of the diagnostic criteria used in different studies. The Rome IV criteria^5^ are currently the most widely accepted method for diagnosing FGIDs.

The pathophysiology of FGIDs is still not fully established.^6^ It has been proposed that interaction of multiple factors, such as genetic susceptibility, early life experiences, sociocultural issues and coping mechanisms, leads to development of different phenotypes and pain response behaviors, including FGIDs.

 Prematurity is considered to be a risk factor for the development of some FGIDs, especially infant colic^7^ and regurgitation.^8^ A recent study^9^ using the Rome III criteria showed that there was higher prevalence of FGIDs among infants born preterm than among infants born full term; however, both groups showed high prevalence of FGIDs. Recent studies conducted in Colombia and Europe have found no association between prematurity and functional gastrointestinal disorders, respectively, in the age ranges of 10 to 18 years and under 4 years.^10^ ^,^ ^11^

In general, preterm infants face greater complications during the neonatal period, such as longer hospital stays, greater need for invasive painful procedures and higher use of medications, which may lead to changes to the development and maturation of some organs, such as the brain and intestines; these changes might explain abnormalities in pain processing^12^ and thus contribute to the onset of FGIDs.

## OBJECTIVE

The main objective of this study was to compare the frequency of FGIDs in infants born preterm with that in infants born at term, using the Rome IV criteria. The secondary objective was to evaluate whether there was any association between sociodemographic variables and invasive procedures during the neonatal period and the development of FGIDs in preterm infants during their infancy.

## METHODS

### Study design

This was a controlled nested cross-sectional study in a retrospective cohort of premature infants who were compared with a group of full-term babies, with regard to the presence of FGIDs.

All infants in both groups underwent a consultation with a pediatric gastroenterologist. In the consultation, information on the infant’s gestational and neonatal history and any occurrence of gastrointestinal symptoms was obtained, and a complete physical examination was performed. The Rome IV criteria were used to characterize FGIDs.

Infants were included after their guardians had agreed to participate in the study and had signed an informed consent statement. The project was approved by the Research Ethics Committee of the Universidade Federal de São Paulo (UNIFESP) under CAAE no. 66233517.0.0000.5505, on May 5, 2017.

### Preterm and term groups

All preterm infants seen consecutively at the Preterm Outpatient Clinic of the Neonatal Pediatric Division of the Department of Pediatrics of Escola Paulista de Medicina (EPM), UNIFESP, during the study period (2017-2019), were recruited. The following inclusion criteria were adopted: gestational age less than 37 weeks and current corrected age at the time of the study of between 30 days and 24 months.

The group of full-term infants (aged 30 days to 24 months) was recruited from a primary care unit and an immunization center, both in the metropolitan region of São Paulo. Infants born at a gestational age greater than or equal to 37 weeks and a birth weight > 2500 g were included.

The following exclusion criteria were used for both groups: previous abdominal surgery, presence of past or current gastrointestinal tract disease, cerebral palsy, severe congenital malformations and presence of an alternative feeding route (such as gastrostomy or enteral probe) or tracheostomy.

### Gestational and neonatal history and economic classification

Records containing information on the cohort of preterm infants since their birth are kept at the Preterm Outpatient Clinic of the Neonatal Pediatric Division of Escola Paulista de Medicina (EPM). Thus, data were collected regarding the gestational period (parity and age of the mother and complications during pregnancy) and neonatal period (gestational age, weight and length at birth, type of delivery, neonatal complications and type of feeding started in the maternity ward). The weight-for-gestational age was classified in accordance with the INTERGROWTH-21^st^ curve.^14^ Data on invasive procedures performed during the stay in the neonatal unit (orotracheal intubation, umbilical catheterization or use of orogastric tube), any use of red blood cell transfusion, parenteral nutrition, cardiopulmonary resuscitation in the delivery room or antibiotics in the neonatal period, the length of hospital stay and any occurrence of sepsis were recorded.

For the full-term infants, these data were obtained from the maternity discharge summary in the child’s health record or, in the absence of these documents, from information provided by the mother or guardian.

Socioeconomic class was evaluated based on the points system of the Brazil Criterion version 2015 of the Brazilian Association of Survey Companies (Associação Brasileira de Empresas de Pesquisa, ABEP; São Paulo, Brazil).

### Standardized consultation with pediatric gastroenterologist

Pediatric consultations for the infants in both groups were conducted by a single pediatric gastroenterologist. The frequency of regurgitation and its warning signs (retching, hematemesis, aspiration, apnea, failure to thrive, feeding or swallowing difficulties or abnormal posturing), daily duration of crying and irritability, presence and duration of efforts to evacuate, frequency of evacuation, stool consistency, presence of pain when evacuating and any history of fecal impaction were recorded. Considering these clinical characteristics, the diagnosis of FGIDs was established in accordance with the Rome IV criteria^5^ for infant regurgitation, infant colic, functional diarrhea, infant dyschezia and functional constipation. To ensure greater accuracy of the results and avoid memory bias, only the clinical gastrointestinal tract manifestations that occurred at the time of the study were considered. FGIDs that had occurred in the past and had disappeared by the time of the study were not considered.

During the consultation with the pediatric gastroenterologist, the following information was also collected: history of previous infection (acute otitis media, bronchiolitis, pneumonia, urinary tract infection or acute gastroenteritis); family history of gastrointestinal disorders; current type of breastfeeding (breast milk, infant formula or cow’s milk); and current use of medications (use of ranitidine, domperidone, anticonvulsants, antibiotics, prednisolone and inhaled corticosteroids was investigated).

Regarding the family history of gastrointestinal disorders, information was collected about any family history (among parents and first-degree siblings) of chronic constipation, abdominal pain or gastroesophageal reflux.

A physical examination was performed, and the results, including weight and length, were recorded. Weight was measured using a Welmy digital pediatric scale (Welmy, Santa Bárbara d’Oeste, Brazil) among the term infants; and using a Filizola Baby digital pediatric scale (Filizola, São Paulo, Brazil) among the preterm infants. Length was measured using a child stadiometer (0-99 cm). Weight-for-age, height-for-age and body mass index-for-age Z scores were calculated using the Anthro software, version 3.2.2 (World Health Organization, Geneva, Switzerland).

### Statistical analysis

The qualitative variables and occurrence of FGIDs in both groups (preterm and full-term infants) were compared using the chi-square test or Fisher’s exact test. Regarding the quantitative variables, Student’s t test was used to compare the mean ± standard deviation when the data were normally distributed; otherwise, the median and first and third quartiles were compared using the Mann-Whitney test. When necessary, a logistic regression model adjusted for age was used. The results from the logistic regression were presented as the odds ratio (OR) and respective 95% confidence interval (95% CI).

All analyses were performed using STATA/SE 15.1 for Windows (StataCorp, College Station, Texas, United States). P-values < 0.05 were considered statistically significant.

## RESULTS

The study included 103 infants who were born preterm. Among these, four were excluded because they had a gastrointestinal tract disease (one had gastroesophageal reflux disease, one had cow’s milk protein allergy and two had lactose intolerance, according to information provided by their mothers). The group of infants born full term consisted of 99 infants, and one was excluded due to gastroesophageal reflux disease. Thus, 99 infants born preterm and 98 born full term were studied. In the preterm group, the current corrected age was used.

Table 1 shows the characteristics of the infants and their gestational and neonatal histories. The corrected age of the preterm infants was significantly higher than that of the term infants. There was a higher proportion of male infants in the preterm infant group, but the difference was not significant. The mean gestational age was 31.4 weeks for the preterm infants and 39.2 weeks for the term infants. The preterm group had lower birth weight and length and higher proportions of cesarean delivery, need for resuscitation in the delivery room and small-for-gestational-age (SGA) infants, and these differences were significant. A higher rate of exclusive breastfeeding in the maternity ward was observed for the infants who were born full term. There were also significant differences in the current weight-for-age, length-for-age and body mass index-for-age Z scores between the groups. Most of the infants in both groups belonged to social classes C, D or E. Preterm infants had higher rates of previous infections and were currently using more medications and consuming less breast milk than full-term infants.

**Table 1.** Characteristics and histories of the preterm and term infants

	Preterm (n = 99)	Term (n = 98)	P
Age (months)	7.2 (3.6; 13.2)	5.3 (3.1; 10.6)	0.010^a^
Sex (M/F)	58/41	48/50	0.176^b^
Gestational age (weeks)	31.4 ± 2.6	39.2 ± 1.0	< 0.001^c^
Weight at birth (g)	1,404.2 ± 379.8	3,321.9 ± 402.8^e^	< 0.001^c^
Length at birth (cm)	38.5 ± 3.4	48.9 ± 2.0^f^	< 0.001^c^
Cesarean delivery	76.8%	46.4%^g^	< 0.001^b^
Small for gestational age	33.3%	12.4%^h^	0.001^b^
Resuscitation in the delivery room	43.4%	2.2%^i^	< 0.001^b^
Antibiotic use in the neonatal period	96.9%^j^	0.0%	< 0.001^b^
Exclusive breastfeeding in the maternity ward	0.0%	84.4%^e^	< 0.001^d^
Mixed feeding in the maternity ward	89.9%	15.6%^e^	< 0.001^d^
Current weight-for-age Z score	-0.670	-0.185	< 0.001^a^
Current length-for-age Z score	-0.940	-0.510	0.002^a^
Current BMI-for-age Z score	-0.32	+0.33	< 0.001
Maternal disease during pregnancy	33.3%	14.4%^g^	0.002^b^
Socioeconomic classes C, D and E	86.8%	71.7%^l^	0.016
Current feeding type			
Breast milk	13.1%	79.4%^g^	< 0.001^b^
Infant formula	74.7%	37.1%^g^	< 0.001^b^
Cow’s milk	25.3%	17.5%^g^	0.253
Personal history of infection	52.5%	28.9% ^g^	0.003^b^
Current use of medications	40.4%	4.1% ^g^	< 0.00^b^
Family history of gastrointestinal disorders	31.3%	24.5%^m^	0.566^b^

M = male; F = female. ^a^Mann-Whitney test; ^b^chi-square test; ^c^Student’s t test; ^d^Fisher’s exact test; ^e-m^information available from the following numbers of infants: ^e^n = 96; ^f^n = 91; ^g^n = 97; ^h^n = 89; ^i^n = 90; ^j^n = 65; ^l^n = 92; ^m^n = 98.

Table 2 shows the frequency of FGIDs in both groups. The number of infants evaluated for each disorder varied according to the age established through the Rome IV criteria: infant colic, up to 5 months; regurgitation, up to 12 months; dyschezia, up to 9 months; constipation, up to 24 months; and functional diarrhea, between 6 and 24 months.

**Table 2.** Functional gastrointestinal disorders among preterm and term infants according to the Rome criteria, considering age ranges

Disorder (with age range)	Preterm	Term	P
	% (n/N)	% (n/N)	
Infant regurgitation			
(21 days to 12 months)	15.6% (10/64)	35.1% (26/74)	< 0.001^a^
Infant colic			
(birth to 5 months)	10.7% (3/28)	4.3% (2/46)	0.360^b^
Infant dyschezia			
(birth to 9 months)	9.3% (4/43)	7.5% (5/67)	0.735^b^
Constipation			
(birth to 24 months)	17.2% (17/99)	9.2% (9/98)	0.098^a^
Functional diarrhea			
(6 to 24 months)	4.6% (3/65)	0.0% (0/43)	0.274^b^
One or more FGIDs			
(1 to 8 months)	37.8% (17/45)	47.8% (32/67)	0.298^a^
(9 to 12 months)	36.8% (7/19)	14.3% (1/7)	0.375^b^
(13 to 24 months)	20% (7/35)	16.7% (4/24)	0.747^a^

^a^Chi-square test; ^b^Fisher’s exact test. FGIDs = functional gastrointestinal disorders.

The frequency of infant regurgitation was higher in term infants than in preterm infants (Table 2). Among the infants in the age group for the evaluation of regurgitation, it was observed that the mean age of the term infants (4.7 ± 2.6 months) was lower (P = 0.01) than that of the preterm infants (6.0 ± 3.5 months). In turn, the proportion of infants who received breast milk was higher (P < 0.001) in the full-term group than in the preterm infant group. Considering the differences between the groups regarding age and proportion of breastfeeding, a multivariate analysis was performed (Table 3) with adjustment for these factors. Infant regurgitation remained associated with full-term birth and younger age.

**Table 3.** Bivariate and multivariate analyses on the odds of infant regurgitation according to the Rome IV criteria, adjusted for breastfeeding and infant age

Factor	OR_crude_	95% CI	P-value	OR_adjusted_	95% CI	P-value
Group						
(term versus preterm)	2.93	1.28-6.68	0.011	3.85	1.26-11.78	0.018
Age, months	0.78	0.67-0.91	0.001	0.76	0.63-0.91	0.002
Breastfeeding	1.77	0.82-3.84	0.149	0.50	0.16-1.51	0.219

OR = odds ratio; CI = confidence interval.

For regurgitation, we also did a separate analysis according to semester for the two groups, with the following results. Regarding regurgitation among infants < 6 months, 7/34 (18.9%) of the premature infants and 25/55 (45.6%) of the term infants had regurgitation. When analyzed using the chi-square test, we found a statistical difference between the groups, with P = 0.032, i.e. those born at term continued to present a higher prevalence of regurgitation even when analyzed according to semester. Regarding regurgitation among infants > 6 months, this was present in 3/30 (10%) of the preterm infants and 1/19 (5.53%) of the term infants. As expected, the prevalence of regurgitation in the second age category was low in both groups.

Despite the higher frequencies of infant colic, functional constipation, functional diarrhea and infant dyschezia in the group of preterm infants, the differences were not significant. When the presence of at least one FGID was evaluated, no difference was observed between the groups (Table 2).

In the group of preterm infants, the association between sociodemographic variables and neonatal factors (including invasive procedures) and FGIDs was studied. The factors of sex, method of delivery, gestational age, birth weight, SGA, resuscitation in the delivery room, orotracheal intubation, arterial or venous umbilical catheterization, use of an orogastric tube, blood transfusion, use of parenteral nutrition, presence of sepsis, use of antibiotic in the neonatal period and length of hospital stay did not show any relationships (P > 0.05) with occurrences of infant regurgitation, infantile colic, functional diarrhea, dyschezia or constipation.

When necessary, the guardians of infants with an FGID were instructed by the researcher regarding specific therapeutic measures. Infants with more severe clinical manifestations were referred for evaluation and follow-up at the outpatient clinic of the Pediatric Gastroenterology Division of UNIFESP.

## DISCUSSION

In our study, higher frequency of FGIDs among preterm infants or those with a history of invasive neonatal procedures was not observed. A recent American study^13^ using the Rome IV criteria and based on data collected online from 58 infants in the first year of life and 238 children between one and three years of age found prevalences of FGIDs that were similar to ours: 24.1% for infant regurgitation, 5.2% for infant colic and 12.0% for constipation. None of the infants had functional dyschezia or functional diarrhea. Our results provide evidence compatible with that of other studies in the literature,^13^ ^,^ ^15^ ^,^ ^16^ but in addition, it is one of the few studies based on information obtained during face-to-face consultations performed specifically for this purpose by a pediatric gastroenterologist.

There are few studies in the literature comparing the prevalence of FGIDs among infants born preterm with that of infants born full term. A prospective study^9^ on Italian infants born preterm or at term showed that during the first year of life, occurrence of at least one FGID according to the Rome III criteria was more prevalent among preterm infants (86%) than among term infants (73%). The high cumulative prevalence of at least one FGID in that study, compared with the findings in our study and in reviews of the literature is noteworthy.^4^ ^,^ ^15^ Preterm infants had higher prevalence of regurgitation (45.7%) than term infants (37.3%; P < 0.015), in contrast with the results obtained in our study. In our study, regurgitation occurred in 35.1% of term infants and in 15.6% of preterm infants (P < 0.001). This difference could be explained by the type of feeding, since infants fed with maternal milk may present more regurgitation.^17^

However, the multivariate analysis (Table 3) showed that the greater frequency of breastfeeding in the group of term infants did not explain the higher frequency of regurgitation. In the Italian study,^9^ no relationship was found between the type of feeding and infant regurgitation. The age difference between the two groups may be one of the factors explaining the higher prevalence of regurgitation among infants born at term. However, according to this multiple regression analysis, the greater chance of regurgitation among full-term infants was maintained even after adjusting for age (odds ratio, OR = 3.85; 95% confidence interval, CI: 1.26; 11.78; P = 0.018) (Table 3).

Although there was no significant difference in the frequency of infant colic in our study, it was higher among preterm infants. This was also observed in the Italian study.^9^ However, the high cumulative rate of infant colic (more than 40% of the study population) in that study is noteworthy. This rate was higher than what was found in a Danish study^7^ on a cohort of 62,761 infants that was based on the results from a computer-assisted telephone interview. In that study, prevalences of colic of 7.6% among term infants and 10.7% among preterm infants were found. These values were similar to those found in our study.

The higher frequency of infant colic among preterm infants may be related to changes in gastrointestinal motility, lower production of gastric acid and proteolytic enzymes and lower concentrations of secretory immunoglobulin A (IgA) and antimicrobial peptides in the gastrointestinal tract, which increase the risk of dysbiosis.^18^ Preterm infants also have lower concentrations of lactase in the intestinal villi, compared with term infants.^18^ Increased lactose concentration in the large intestine may lead to higher production of lactic acid and gases, which can cause pain and abdominal distension. Both dysbiosis^19^ and lactase deficiency^20^ are possible factors related to the development of infant colic.

The recent observation that use of antibiotics may cause predisposition to dysbiosis and, thus, to greater odds of developing FGIDs may be included in this context. In our study, we reviewed data on antibiotic use in the neonatal unit: among the 63 preterm infants who used antibiotics during the neonatal period, 21 (33.3%) developed FGID. Neither of the two preterm infants who did not use antibiotics developed any FGID. We used Fisher’s exact test to analyze these data and did not find any relationship between antibiotic use during the neonatal period in the preterm group and future development of any FGID (P = 1.00).

We found higher frequency of constipation among preterm infants than among term infants (17.2% versus 9.2%), but the difference was not significant (P = 0.098). It is worth noting that for a significant difference to be achieved (80% power and 5% alpha error), it would have been necessary to include 361 patients in each group, which would have been unfeasible from an operational point of view. A study conducted in Denmark^17^ among 286 preterm infants showed a high rate (approximately 40%) of laxative use among preterm infants up to six months of age.

The only study^9^ in the literature that evaluated functional diarrhea among preterm infants showed a prevalence of 3.3%, similar to what was observed in our study. There was also no difference between full and preterm infants. A study conducted in Latin America^21^ found that the prevalence of functional diarrhea was 1.9% among infants in the first year of life, regardless of gestational age at birth.

Regarding dyschezia, the findings from our study also contradicted the expectation of higher frequency among preterm infants: the rates found were 9.3% among preterm infants and 7.5% among term infants (P = 0.735). Dyschezia is a functional disorder that has been little investigated, with few studies in the literature. It results from lack of coordination between increased abdominal pressure and pelvic floor relaxation during bowel movements.^5^ It has been assumed that preterm infants may have a delay in this mechanism that leads to higher rates of dyschezia. However, we were unable to confirm this hypothesis.

We also investigated whether neonatal factors in the group of preterm infants, including invasive diagnostic and therapeutic procedures that were experienced during the neonatal period, could contribute to development of FGIDs. Orotracheal intubation, use of venous or arterial umbilical catheters, use of an orogastric tube, parenteral nutrition, length of hospital stay, type of delivery, history of sepsis, gestational age, birth weight and weight for gestational age classification were some of the factors analyzed. Although it has been suggested in the literature^7^ that these factors could alter the maturation and development of some organs in newborns, thus leading to greater risk of development of FGIDs, we were unable to confirm this relationship in our study.

Our study had some limitations, such as the small number of infants included in the independent analysis on each of the five FGIDs, according to the age ranges recommended through the Rome IV criteria. For example, we included a total of 99 preterm infants in our study, but only 64 were analyzed regarding the presence of regurgitation (infants up to 12 months of age). Moreover, the number of preterm infants with FGIDs was small, which may have compromised the analysis on the relationship between invasive diagnostic and therapeutic procedures and the development of FGIDs. Other limitations were the cross-sectional nature of the study, which did not allow characterization of all FGIDs that had occurred or would occur during the first two years of life, and our use of a sample consisting of all patients consecutively treated at the outpatient clinic, which prevented calculation of the prevalence in a probabilistic sample.

On the other hand, the strength of our study was that it was the first to compare the frequency of FGIDs among preterm infants with their frequency among full-term infants using a comparative design and face-to-face consultations in which the Rome IV criteria were used. These face-to-face consultations decreased the risk of understanding bias, which can occur in studies that use only questionnaires. A physical examination was also performed on all the infants, which enabled a more complete assessment that, in addition to inclusion of weight and length data, helped establish the diagnosis of FGIDs.

## CONCLUSIONS

We did not find any evidence of higher frequency of FGIDs among preterm infants than among infants born full term, during the first two years of life. In contrast, we found higher frequency of regurgitation among infants born full term than among preterm infants. In addition, there was no relationship between invasive diagnostic and therapeutic procedures performed during the neonatal period among infants born preterm and development of FGIDs during infancy.

## References

[1] Rasquin-Weber A, Hyman PE, Cucchiara S (1999). Childhood functional gastrointestinal disorders. Gut.

[2] Dhroove G, Chogle A, Saps M (2010). A million-dollar work-up for abdominal pain: is it worth it?. J Pediatr Gastroenterol Nutr.

[3] Vandenplas Y, Hauser B, Salvatore S (2019). Functional Gastrointestinal Disorders in Infancy: Impact on the Health of the Infant and Family. Pediatr Gastroenterol Hepatol Nutr.

[4] Vandenplas Y, Abkari A, Bellaiche M (2015). Prevalence and Health Outcomes of Functional Gastrointestinal Symptoms in Infants From Birth to 12 Months of Age. J Pediatr Gastroenterol Nutr.

[5] Benninga MA, Faure C, Hyman PE (2016). Childhood Functional Gastrointestinal Disorders: Neonate/Toddler. Gastroenterology.

[6] Bonilla S, Saps M (2013). Early life events predispose the onset of childhood functional gastrointestinal disorders. Rev Gastroenterol Mex.

[7] Milidou I, Søndergaard C, Jensen MS, Olsen J, Henriksen TB (2014). Gestational age, small for gestational age, and infantile colic. Paediatr Perinat Epidemiol.

[8] Eichenwald EC, Committee on Fetus and Newborn (2018). Diagnosis and Management of Gastroesophageal Reflux in Preterm Infants. Pediatrics.

[9] Salvatore S, Baldassarre ME, Di Mauro A (2019). Neonatal antibiotics and prematurity are associated with an increased risk of functional gastrointestinal disorders in the first year of life. J Pediatr.

[10] Velasco-Benitez CA, Axelrod CH, Gutierrez S, Saps M (2020). The relationship between prematurity, method of delivery, and functional gastrointestinal disorders in children. J Pediatr Gastroenterol Nutr.

[11] Steutel NF, Zeevenhooven J, Scarpato E (2020). Prevalence of Functional Gastrointestinal Disorders in European Infants and Toddlers. J Pediatr.

[12] Miranda A (2008). Early life events and the development of visceral hyperalgesia. J Pediatr Gastroenterol Nutr.

[13] Villar J, Ismail LC, Victora CG (2014). International standards for newborn weight, length, and head circumference by gestational age and sex; the Newborn Cross-Sectional Study for the INTERGROWTH-21st Project. Lancet.

[14] Robin SG, Keller C, Zwiener R (2018). Prevalence of Pediatric Functional Gastrointestinal Disorders Utilizing the Rome IV Criteria. J Pediatr.

[15] Ferreira-Maia AP, Matijasevich A, Wang YP (2016). Epidemiology of functional gastrointestinal disorders in infants and toddlers: A systematic review. World J Gastroenterol.

[16] Russo M, Strisciuglio C, Scarpato E (2019). Functional Chronic Constipation: Rome III Criteria Versus Rome IV Criteria. J Neurogastroenterol Motil.

[17] Zachariassen G, Fenger-Gron J (2014). Preterm dietary study: meal frequency, regurgitation and the surprisingly high use of laxatives among formula-fed infants following discharge. Acta Paediatr.

[18] Lenfestey MW, Neu J (2018). Gastrointestinal Development: Implications for Management of Preterm and Term Infants. Gastroenterol Clin North Am.

[19] de Weerth C, Fuentes S, Puylaert P, de Vos WM (2013). Intestinal microbiota of infants with colic: development and specific signatures. Pediatrics.

[20] Ahmed M, Billoo AG, Iqbal K, Memon A (2018). Clinical Efficacy Of Lactase Enzyme Supplement In Infant Colic: A Randomised Controlled Trial. J Pak Med Assoc.

[21] Chogle A, Velasco-Benitez CA, Koppen IJ (2016). A Population-Based Study on the Epidemiology of Functional Gastrointestinal Disorders in Young Children. J Pediatr.

